# E2F4 Promotes Neuronal Regeneration and Functional Recovery after Spinal Cord Injury in Zebrafish

**DOI:** 10.3389/fphar.2016.00119

**Published:** 2016-05-09

**Authors:** Shota Sasagawa, Yuhei Nishimura, Yuka Hayakawa, Soichiro Murakami, Yoshifumi Ashikawa, Mizuki Yuge, Shiko Okabe, Koki Kawaguchi, Reiko Kawase, Toshio Tanaka

**Affiliations:** ^1^Department of Molecular and Cellular Pharmacology, Pharmacogenomics and Pharmacoinformatics, Mie University Graduate School of MedicineTsu, Japan; ^2^Mie University Medical Zebrafish Research CenterTsu, Japan; ^3^Department of Systems Pharmacology, Mie University Graduate School of MedicineTsu, Japan; ^4^Department of Omics Medicine, Mie University Industrial Technology Innovation InstituteTsu, Japan; ^5^Department of Bioinformatics, Mie University Life Science Research CenterTsu, Japan

**Keywords:** spinal cord injury, systems pharmacology, comparative transcriptome analysis, zebrafish, E2F4, DREAM complex

## Abstract

Mammals exhibit poor recovery after spinal cord injury (SCI), whereas non-mammalian vertebrates exhibit significant spontaneous recovery after SCI. The mechanisms underlying this difference have not been fully elucidated; therefore, the purpose of this study was to investigate these mechanisms. Using comparative transcriptome analysis, we demonstrated that genes related to cell cycle were significantly enriched in the genes specifically dysregulated in zebrafish SCI. Most of the cell cycle-related genes dysregulated in zebrafish SCI were down-regulated, possibly through activation of e2f4. Using a larval zebrafish model of SCI, we demonstrated that the recovery of locomotive function and neuronal regeneration after SCI were significantly inhibited in zebrafish treated with an E2F4 inhibitor. These results suggest that activation of e2f4 after SCI may be responsible, at least in part, for the significant recovery in zebrafish. This provides novel insight into the lack of recovery after SCI in mammals and informs potential therapeutic strategies.

## Introduction

Spinal cord injury (SCI) in mammals typically results in permanent neurological deficits, whereas regenerative organisms, such as amphibians and fish, are capable of regeneration after SCI (reviewed in Lee-Liu et al., 2013; Vajn et al., 2013; Silver et al., 2015). Certain key differences between regenerative and non-regenerative organisms have been identified. For example, in regenerative organisms, astroglial cells form a bridge, and aid axonal elongation after injury. In contrast, after SCI in mammals astrocytes form a glial scar and secrete extracellular matrix (ECM) components such as chondroitin sulfate and proteoglycans. These extrinsic mechanisms can cause a relative lack of growth-promoting molecules and/or a surplus of growth-inhibitory molecules, which can result in the inhibition of axonal regeneration in mammalian SCI. It has also been demonstrated that the intrinsic propensity for axonal growth is different between regenerative and non-regenerative organisms. For example, the expression of miR-133b was increased at 1 and 7 days after SCI in zebrafish and knockdown of miR-133b significantly attenuated axonal regeneration (Yu et al., 2011). In contrast, the expression of miR-133b was transiently increased at 4 h after SCI and dramatically decreased at 1 day after SCI in rat (Liu et al., 2009). There are several targets of miR-133b that are involved in regeneration after SCI (Vajn et al., 2013). The balance between extrinsic and intrinsic molecular events may determine the overall axonal regeneration response. A comprehensive understanding of the mechanisms underlying the difference between non-regenerative and regenerative organisms is therefore still required.

Transcriptome analysis using microarrays and high-throughput sequencing have provided new insights into the pathogenesis of various disorders, including SCI in mouse (Chen et al., 2013; Wu et al., 2013), rat (Di Giovanni et al., 2003; Chamankhah et al., 2013; Chen et al., 2015), and zebrafish (Ma et al., 2012; Hui et al., 2014). These studies included time-series expression analysis; the clustering of differentially expressed genes (DEGs) based on their temporal expression patterns. This approach has revealed molecular events occurring in the acute, subacute, and chronic stage of SCI. Recently, genome-wide expression profiling was performed to compare the response to SCI in *Xenopus laevis* at regenerative and non-regenerative stages (Lee-Liu et al., 2014). The analysis revealed that very different sets of transcripts are produced in the regenerative and non-regenerative stages after SCI.

These findings suggest that there might be differences in the DEGs between regenerative and non-regenerative organisms after SCI and that there might be transcription factors (TFs) regulating the DEGs that might be selectively activated or inhibited in either regenerative or non-regenerative organisms. To identify these TFs, we applied systems biology approaches to public transcriptome data for SCI in zebrafish (Hui et al., 2014), mouse (Wu et al., 2013), and rat (Chamankhah et al., 2013). We were able to identify several TFs selectively functioning in zebrafish SCI or mouse/rat SCI. We were also able to demonstrate that e2f4, a member of the DREAM complex (TF**D**P1, **R**BL2, **E**2F4, **a**nd **M**uvB core complex) and a master coordinator of cell cycle dependent gene transcription (Sadasivam and DeCaprio, 2013), was selectively activated in zebrafish SCI and promoted neuronal regeneration and functional recovery.

## Materials and methods

### Ethics statement

This study was carried out in strict accordance with Japanese law [The Humane Treatment and Management of Animals (2014)^1^, Standards Relating to the Care and Management of Laboratory Animals and Relief of Pain (2013)^2^ and the Guidelines for Proper Conduct of Animal Experiments (Science Council of Japan, 2006)^3^]. All surgery was performed under 2-phenoxyethanol anesthesia, and all efforts were made to minimize suffering.

### Compounds

HLM006474 was obtained from Tocris (Bristol, UK). A stock solution of HLM006474 was prepared by dissolving in dimethyl sulfoxide (Nacalai Tesque, Kyoto, Japan). 2-phenoxyethanol was obtained from Wako Chemical (Osaka, Japan).

### Comparative transcriptome analysis

To compare DEGs among mouse, rat, and zebrafish SCI, we used three transcriptome data sets deposited in the Gene Expression Omnibus (GEO; Barrett et al., 2009). In the mouse SCI model (Wu et al., 2013), T9 was injured by contusion with an impactor. In the rat SCI model (Chamankhah et al., 2013), T7 was injured by compression with a clip. In the zebrafish SCI model (Hui et al., 2014), the 15/16th vertebrae was injured by crushing dorso-ventrally with forceps. In these models, the spinal cord containing the epicenter of the injured tissues was extracted for the transcriptome analysis. The raw transcriptome analysis data of mouse SCI (GSE47681) (Wu et al., 2013), rat SCI (GSE45006) (Chamankhah et al., 2013), and zebrafish SCI (GSE39295) (Hui et al., 2014) were downloaded from GEO (Barrett et al., 2009). The raw data were normalized using “affy” (Gautier et al., 2004) for GSE47681 and GSE45006 or “limma” (Ritchie et al., 2015) for GSE39295 in Bioconductor (Gentleman et al., 2004). Probes with reliable signals were selected and subjected to “RankProd” (Hong et al., 2006) to identify DEGs in SCI compared to sham in each model using a false discovery rate of 20% as the threshold. The gene symbols of DEGs in each model were converted to those of human orthologous genes using Life Science Knowledge Bank (World Fusion, Tokyo, Japan). SwissProt IDs of the human orthologous genes were added using BioMart (Smedley et al., 2015). The list of DEGs is shown in Tables S1, S2 for 1 dpi and 3 dpi, respectively. Venn diagrams of the number of DEGs in these models were drawn using “PINA4MS” (Cowley et al., 2012) in Cytoscape (Shannon et al., 2003).

### Identification of enriched gene ontologies in DEGs

To identify enriched gene ontologies in a given gene list, we used DAVID (Huang Da et al., 2009) with medium classification stringency. The clustering algorithm is based on the hypothesis that similar annotations should have similar gene members. The Group Enrichment Score is the geometric mean (in -log scale) of a member's *p*-values in a corresponding annotation cluster. DEGs specific for zebrafish SCI (Tables S1-8, S2-8), DEGs specific for mouse/rat SCI (Tables S1-9, S2-9), DEGs specific for zebrafish SCI potentially regulated by at least two TFs among e2f4, tfdp1 and foxm1 (Tables S5-1,S5-2), and DEGs specific for mouse/rat SCI potentially regulated by Nfic and Tead4 (Table S5-3,S5-4) were subjected to DAVID. The clusters of enriched gene ontologies for these DEGs are shown in Tables S3, S6.

### Identification of transcription factors regulating DEGs using iRegulon

iRegulon exploits the fact that genes that are co-regulated by the same TF commonly share binding sites for the TF and uses gene sets derived from ENCODE ChIP-seq data (Gerstein et al., 2012; Janky et al., 2014). We used iRegulon as an application in Cytoscape (Shannon et al., 2003; Nishimura et al., 2015c; Sasagawa et al., 2016). The lists of DEGs specific for zebrafish SCI (Tables S1-8, S2-8) or mouse/rat SCI (Tables S1-9, S2-9) were subjected to iRegulon using the default settings. The predicted transcriptional regulators with normalized enrichment scores (NES) >3.5 are shown in Table S4.

### Zebrafish strains

We used an albino zebrafish line (Kelsh et al., 1996) obtained from the Max Planck Institute for Developmental Biology (Tübingen, Germany; Watanabe et al., 2012) for locomotive behavior analysis. We also developed Tg(*eno2*: Cerulean) zebrafish. The coding region of Cerulean was amplified by PCR using pCS2+8NCerulean (Addgene, Cambridge, MA, USA) as the template. The PCR product was cloned into Tol2 vector using the In-fusion HD cloning kit (Takara Bio, Shiga, Japan). Briefly, a fragment (from bp 3017 to 1088) of pT2AL200R150G (Kawakami, 2007) was amplified by inverse PCR and fused with the coding region of Cerulean to make a circular plasmid (pT2-cerulean). The promoter of zebrafish *eno2* (from –3783 to 3723 bp) (Bai et al., 2007) was synthesized by Invitrogen (Carlsbad, CA, USA) and cloned into pT2-cerulean using the In-fusion HD cloning kit (Takara Bio) to make pT2-eno2-cerulean. pT2-eno2-cerulean and transposase mRNA were injected into zebrafish embryos at the 1–8 cell stage. Larval zebrafish expressing Cerulean or mCherry in the spinal cord were selected and maintained. Mature F0 zebrafish were mated with albino zebrafish. The F1 zebrafish expressing Cerulean were selected and used for *in vivo* imaging.

Zebrafish were bred and maintained according to previously described methods (Westerfield, 2007; Nishimura et al., 2016). Briefly, zebrafish were raised at 28.5 ± 0.5°C with a 14 h/10 h light/dark cycle. Embryos were obtained via natural mating and cultured in 0.3 × Danieau's solution [19.3 mM NaCl, 0.23 mM KCl, 0.13 mM MgSO_4_, 0.2 mM Ca(NO_3_)_2_, 1.7 mM HEPES, pH 7.2] until 8 dpf for behavioral and *in vivo* imaging analysis. Zebrafish from 5 dpf were maintained on a diet of live Paramecium.

### Microdissection of the zebrafish spinal cord

Zebrafish were anesthetized with 500 ppm 2-phenoxyethanol in 0.3 × Danieau's solution. The probe (EMB-13Y, Nepa Gene, Chiba, Japan), which was connected to a Gastromaster (GST-1, Nepa Gene) through an adaptor (EMB-C125G, Nepa Gene), was placed on the dorsal skin of a zebrafish above the caudal end of the swim bladder. Then, the foot switch was activated using the “yellow” setting of the Gastromaster until complete transection of the spinal cord was made (Movie S1). After surgery, the zebrafish were transferred to a dish containing 0.3 × Danieau's solution without 2-phenoxyethanol. After about an hour, zebrafish were placed individually into wells of a round 48-well-plate (10 mm diameter, containing 500 μL of 0.3 × Danieau's solution). The 48-well-plate was placed in an incubator at 28.5°C until the behavior analysis was performed.

### Assessment of locomotion behavior in zebrafish after SCI

An overview of the behavior analysis is shown in **Figure 3**. All behavioral tests were performed during the same time frame (from 2 to 3 pm at 0, 1, 2, and 3 dpi). A 48-well-plate was placed in a DanioVision observation chamber (Noldus, Wageningen, The Netherlands), which was blocked from daylight and illuminated from below with white light (255 lx) at the time indicated in **Figure 4A**. The locomotive behavior of zebrafish in each well was monitored by the DanioVision system with a resolution of 1024 × 768 pixels at 25 frames per seconds (fps). After assessment of locomotive behavior at 0 dpi, 500 μL of 0.3 × Danieau's solution with or without 5 μM HLM006474 was added to each well of the 48-well-plate and the plate was then placed in an incubator at 28.5°C with constant light (255 lx) from 7 a.m. to 9 p.m. until the behavior analysis was performed the next day. The medium with and without 5 μM HLM006474 was changed after the behavior analysis at 0 dpi. Two independent experiments were performed.

The recorded video images were analyzed using EthoVision XT11 (Noldus) to measure the locomotive behavior of zebrafish in each well. Mobility was calculated by taking every pixel identified as the subject and comparing it between the current image and the previous one (Nishimura et al., 2015b). If all the pixels were the same, there was zero mobility. If all the pixels were different, there was 100% mobility. In this study, we defined 5–35, 35–65, and 65–95% as low, medium, and high mobility. The distance moved by each fish in the dark and light periods was calculated for the time showing medium mobility.

### *In vivo* imaging of zebrafish neurons and astrocytes at the lesion site after SCI

Zebrafish larvae at 3 dpi with or without HLM006474 treatment were anesthetized with 2-phenoxyethanol (500 ppm in 0.3 × Danieau's solution), then transferred onto glass slides. A few drops of 3% low-melting agarose solution were placed over the larvae and the larvae were immediately oriented sideways, dorsal-side-up. The fluorescent signals in the embedded larvae were observed using an epifluorescent microscope (SMZ25, Nikon, Tokyo, Japan) using CFP filters to detect Cerulean fluorescence. Quantitative assessment of the *in vivo* fluorescence imaging was performed using Volocity (Perkin Elmer, Cambridge, MA, USA). Briefly, we placed the region of interest (ROI), a circle 191 μm in diameter, around the SCI lesion (**Figure 5**) in the 256-gray-scale (0–255) image. The area of Cerulean fluorescence in the ROI that contained pixels over the intensity threshold (30) was calculated and was used as the fluorescence signal of neurons.

### Statistical analysis

Statistical analysis was performed using Prism 6 (Graphpad, La Jolla, CA, USA). For the assessment of locomotive behavior, two factor ANOVA followed by Sidak's multiple comparison test was performed. For the assessment of neuronal regeneration, Welch's *t*-test was performed. Data are shown as the mean ± SEM.

## Results

### Comparative transcriptome analysis revealed specific DEGs in zebrafish SCI and mouse/rat SCI

**Figure 1:**
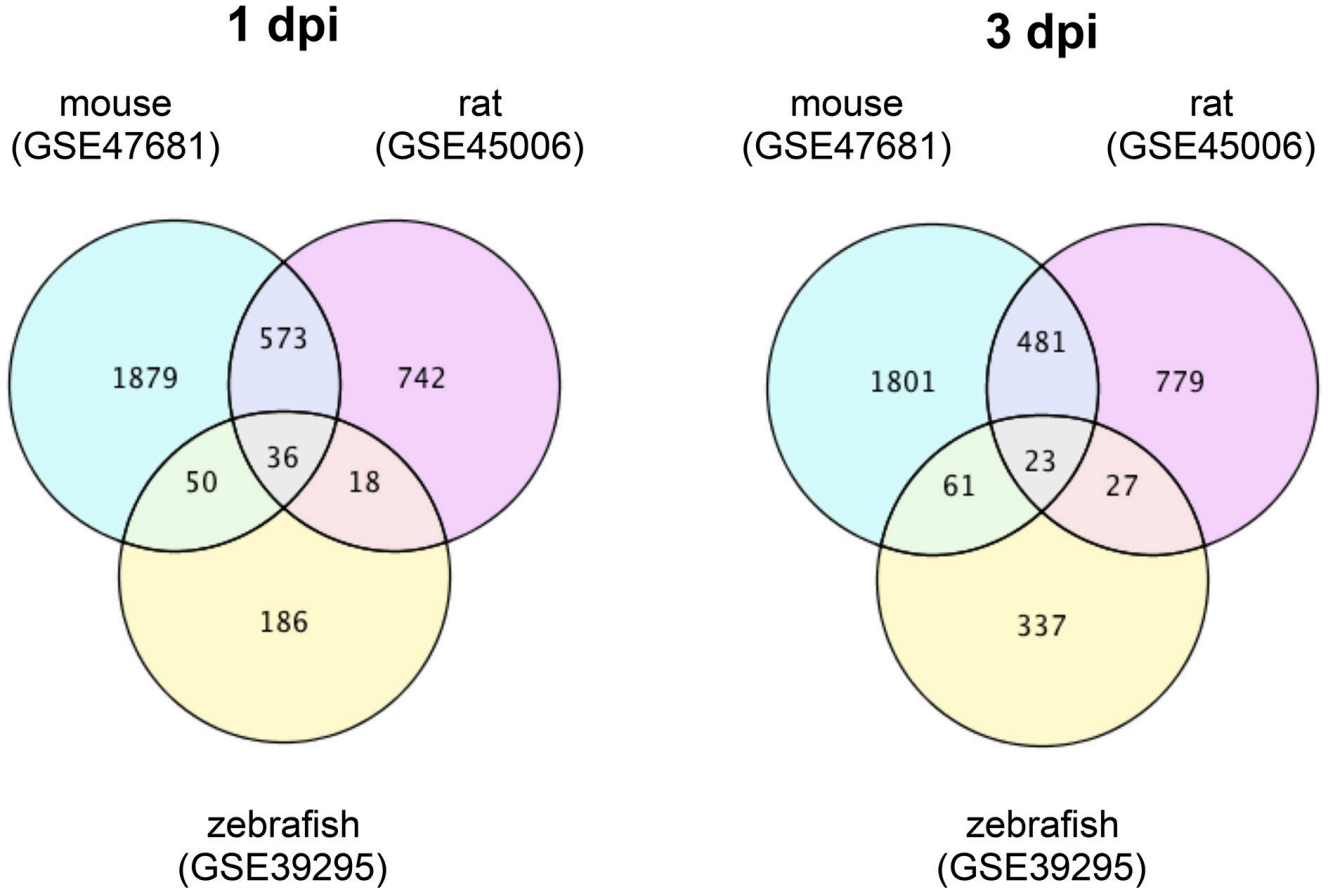
**Venn diagrams of differentially expressed genes in mouse, rat and zebrafish SCI**. Transcriptome data of SCI in mouse (GSE47681), rat (GSE45006), and zebrafish (GSE39295) were downloaded from a public database (GEO). The DEGs between SCI and control groups in each model at 1 or 3 dpi were identified using a false discovery rate of 20% as the threshold. The numbers of DEGs in each group and the overlap between groups are shown in the Venn diagrams.

To identify DEGs that are specific for zebrafish SCI and mouse/rat SCI, we downloaded transcriptome data of mouse SCI (GSE47681) (Wu et al., 2013), rat SCI (GSE45006) (Chamankhah et al., 2013), and zebrafish SCI (GSE39295) (Hui et al., 2014) from the GEO (Barrett et al., 2009). Using these data, we identified DEGs in each SCI model using a false discovery rate of 20% as the threshold. The identified DEGs and their overlap between the three SCI models are shown in Tables S1, S2 for 1 day-post-injury (dpi) and 3 dpi, respectively. Venn diagrams of the numbers of DEGs in these SCI models are shown in Figure 1.

At 1 dpi, 36 DEGs were common among mouse, rat and zebrafish. For 19 of these 36 DEGs, the change in expression was the same in mouse, rat, and zebrafish (Table S1-4), whereas for 17 genes the change in expression was opposite between zebrafish and mouse/rat (Table S1-5). A total of 186 DEGs were identified in zebrafish SCI that were not identified in mouse and/or rat SCI (Table S1-6). Among the 573 DEGs that overlapped between mouse and rat SCI, the change in expression was the same for 553 genes (Table S1-7). In summary, we identified 203 DEGs that were specific for zebrafish SCI (17 DEGs with opposite changes in expression between zebrafish and mouse/rat SCI plus 186 DEGs unique to zebrafish SCI) (Table S1-8) and 570 DEGs that were specific for mouse/rat SCI (17 DEGs with opposite changes in expression between mouse/rat and zebrafish SCI plus 553 DEGs unique to mouse/rat SCI) (Table S1-9).

At 3 dpi, 23 DEGs were common among mouse, rat and zebrafish. Of the 23 DEGs, eight had the same change in expression among mouse, rat and zebrafish (Table S2-4), whereas the expression change of 12 genes was opposite between zebrafish and mouse/rat (Table S2-5). The expression changes of three genes were opposite between mouse and rat (data not shown). In zebrafish SCI, 337 DEGs were identified that were not identified in mouse and/or rat SCI (Table S2-6). Among the 481 DEGs that overlapped between mouse and rat SCI, the changes in expression of 419 genes were the same between mouse and rat (Table S2-7). In summary, we identified 349 DEGs specific for zebrafish SCI (12 DEGs with opposite changes in expression between zebrafish and mouse/rat SCI plus 337 DEGs unique to zebrafish SCI) (Table S2-8) and 431 DEGs specific for mouse/rat SCI (12 DEGs with opposite changes in expression between mouse/rat and zebrafish SCI plus 419 DEGs unique to mouse/rat SCI) (Table S2-9).

### Genes related to cell cycle were enriched in DEGs specific for zebrafish SCI

To reveal the functions of DEGs specific for zebrafish or mouse/rat SCI, we performed gene ontology analysis using DAVID (Huang Da et al., 2009). The analysis revealed that genes related to cell cycle, including mitosis and cyclin, were significantly enriched in DEGs specific for zebrafish SCI at 1 dpi (Table S3-1) and 3 dpi (Table S3-2). Genes related to cell cycle were not enriched in DEGs specific for mouse/rat SCI. Inhibition of cell cycle can stimulate recovery after SCI in mammals (Tian et al., 2007; Wu et al., 2011, 2012). Cell cycle activation after SCI appears to contribute not only to apoptotic cell death of post-mitotic neuronal cells, but also to post-traumatic gliosis and microglial activation (Wu et al., 2011). Therefore, the enrichment of genes related to cell cycle in zebrafish SCI-specific DEGs may be the primary mechanism for neuronal regeneration and functional recovery after SCI in zebrafish, but not in mouse and rat.

### Genes related to cell adhesion were enriched in DEGs specific for mouse/rat SCI

We also revealed that genes related to cell adhesion were significantly enriched in DEGs specific for mouse/rat SCI at 1 dpi (Table S3-3) and 3 dpi (Table S3-4). Genes related to cell adhesion were not enriched in DEGs specific for zebrafish SCI. ECM components, such as chondroitin sulfate, proteoglycans and tanascins are known to be up-regulated after injury and to stimulate cell adhesion and contribute to the growth-inhibitory effects of the glial scar (Busch and Silver, 2007). These findings suggest that cell adhesion may be related to the poor recovery from SCI in mammals.

### Identification of TFs regulating zebrafish SCI-specific DEGs

**Figure 2:**
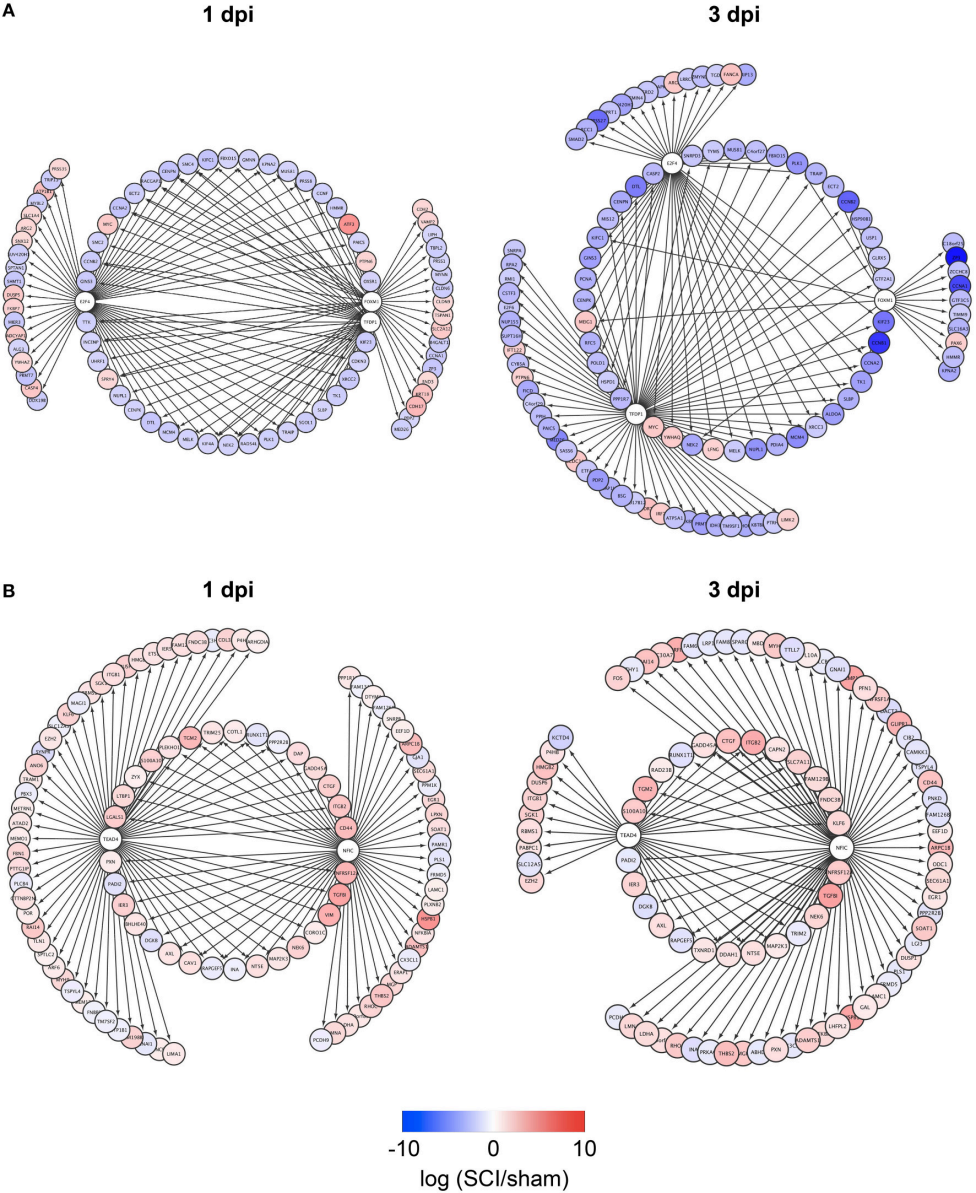
**Identification of e2f4/tfdp1/foxm1 and Nfic/Tead4 as the key TFs that regulate DEGs specific for zebrafish or mouse/rat SCI. (A)** DEGs specific for zebrafish SCI potentially regulated by e2f4, tfdp1 and/or foxm1 at 1 or 3 dpi are shown. **(B)** DEGs specific for mouse/rat SCI potentially regulated by Nfic and/or Tead4 at 1 or 3 dpi are shown.

To identify TFs regulating DEGs specific for zebrafish SCI or mouse/rat SCI, we used iRegulon, a bioinformatics analysis tool that has been successfully used to identify TFs for given gene lists (Janky et al., 2014; Nishimura et al., 2015c; Sasagawa et al., 2016). The analysis identified four and seven TFs potentially regulating DEGs specific for zebrafish SCI at 1 and 3 dpi, respectively (Table S4-1). e2f4, tfdp1, and foxm1 were common among the DEGs at 1 and 3 dpi. Figure 2A demonstrates the genes that were selectively dysregulated in zebrafish SCI, possibly through e2f4, tfdp1, and foxm1. The genes regulated by at least two of e2f4, tfdp1, and foxm1 are shown in the inner circle. Most of the genes in the circle were down-regulated (Tables S5-1,S5-2).

### Identification of TFs regulating mouse/rat SCI-specific DEGs

We also identified TFs potentially regulating DEGs specific for mouse/rat SCI at 1 and 3 dpi (Table S4-2). Two transcriptional activators Nfic (Pjanic et al., 2011) and Tead4 (Jacquemin et al., 1996) were common between the DEGs at 1 and 3 dpi. The DEGs potentially regulated by Nfic and/or Tead4 are shown in Figure 2B. Most of the DEGs were upregulated in mouse/rat SCI, suggesting that both Nfic and Tead4 may be activated in mouse/rat SCI. Genes related to cell adhesion were significantly enriched in the genes potentially regulated by both Nfic and Tead4 at 1 but not 3 dpi (Tables S5-3, S6-3 for 1 dpi, Tables S5-4, S6-4 for 3 dpi). These results suggest that activation of Nfic and Tead4 may contribute to the regulation of DEGs specific for mouse/rat DEG, at least at 1 dpi.

### The recovery of locomotive behavior after SCI was inhibited in zebrafish treated with E2F4 inhibitor

**Figure 3:**
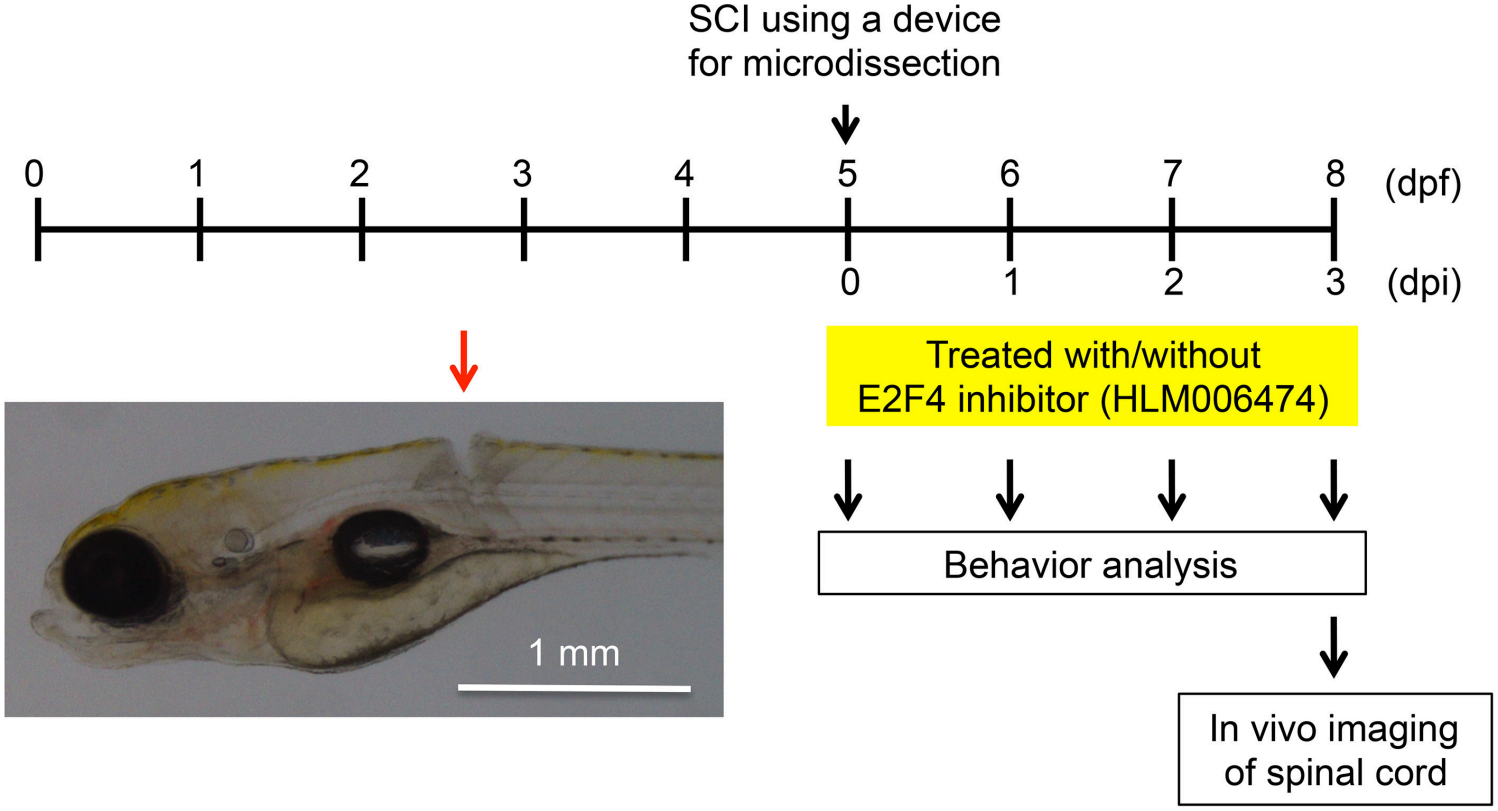
**Larval zebrafish SCI model used in this study**. At 5 dpf, the spinal cords of zebrafish were injured using a device for microdissection (Movie S1). At 0, 1, 2, and 3 dpi, the locomotive behavior of zebrafish was analyzed (Figure 4, Movies S2, S3). At 3 dpi, *in vivo* imaging of the spinal cord was performed (Figure 5). A representative SCI in zebrafish is indicated by the red arrow.

We wanted to examine whether inhibition of e2f4 would affect the recovery of zebrafish locomotive behavior after SCI. In this study, we developed a novel SCI model using larval zebrafish (Figure 3). Using a microdissection device, complete transection of the spinal cord was made above the caudal end of the swim bladder in zebrafish at 5 days post fertilization (dpf; Movie S1).

**Figure 4:**
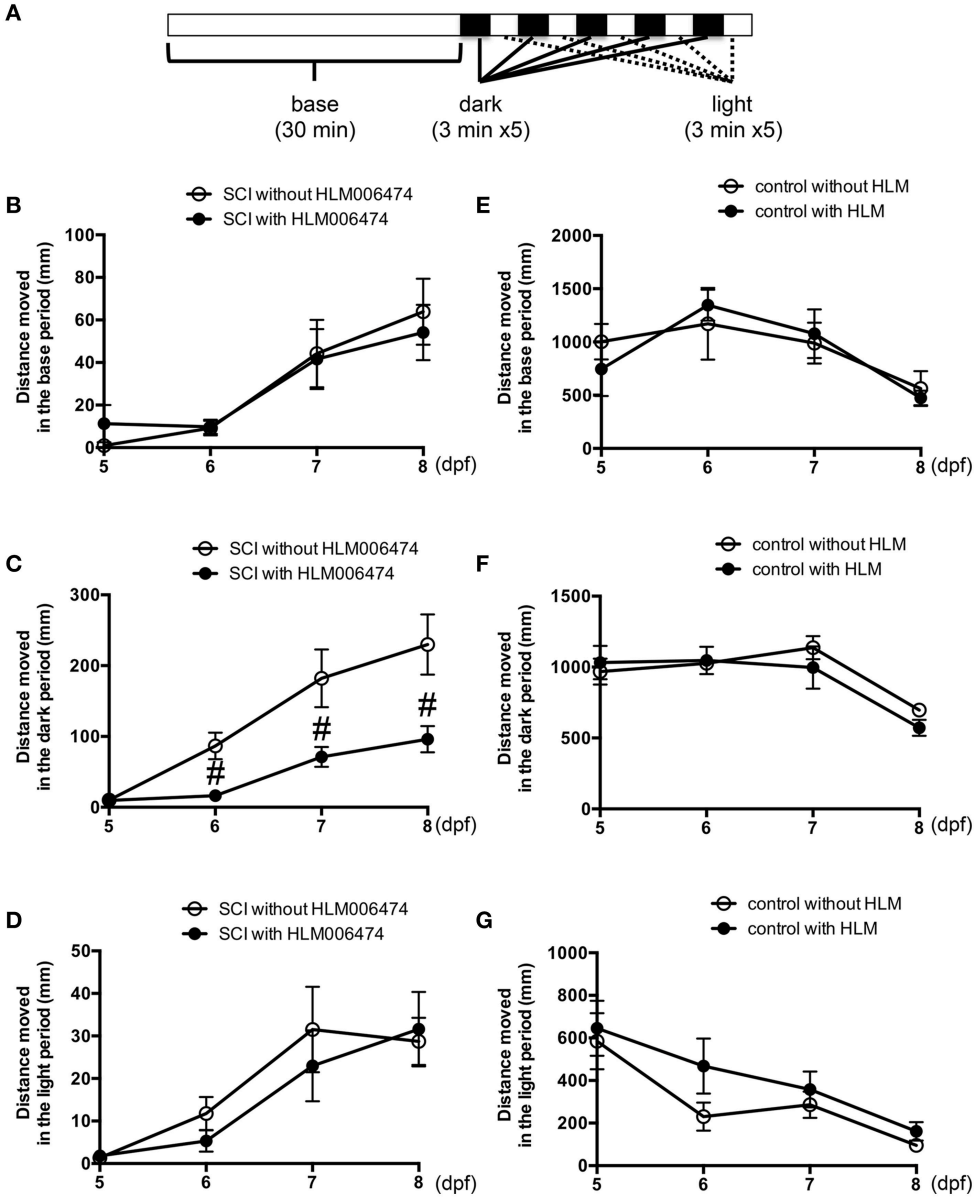
**Recovery of zebrafish locomotive behavior after SCI was inhibited by treatment with E2F4 inhibitor. (A)** Protocol for the assessment of zebrafish locomotive behavior. Zebrafish were placed in each well of a 48-well-plate. The plate was set in a device to monitor the behavior of zebrafish in real-time. In the device, the plate was illuminated for 30 min for acclimatization, followed by five sets of dark (3 min) and light (3 min) periods. Zebrafish with SCI (**B–D**) or without SCI (**E–G**) were treated with an E2F4 inhibitor HLM006474 (5 μM) or with vehicle. The distance moved during the base (30 min, **B,E**), dark periods (15 min, C,F) or light periods (15 min, **D,G**) are shown. *N* = 36 for each group with SCI and eight for each group without SCI. ^#^*p* < 0.05 compared to SCI without HLM006474.

To examine the locomotive ability of zebrafish, we utilized the zebrafish behavior of responding to a change in light/dark conditions during short periods (MacPhail et al., 2009; Nishimura et al., 2015a). The experimental conditions are shown in Figure 4A. The locomotion of zebrafish increases greatly when the conditions change from light to dark (Movie S2), whereas the locomotion decreases when the conditions change from dark to light. Additional cycles of alternating light and dark reliably produce alternating levels of low and high locomotion, respectively (MacPhail et al., 2009). The locomotive behavior of zebrafish with SCI treated with or without HLM006474, an inhibitor of E2F4 (Ma et al., 2008), is shown in Figure 4B–D. The recovery of locomotive behavior during the dark periods was significantly lower in zebrafish treated with HLM006474 (Figure 4C). The locomotive behavior during the base and light periods were not significantly different between zebrafish with and without treatment of HLM006474 (Figures 4B,D). The locomotive behavior of control zebrafish was also not affected by HLM006475 treatment during the base, dark or light periods (Figure 4E–G). These results suggest that inhibition of e2f4 attenuates the functional recovery of zebrafish after SCI.

### Neuronal regeneration after SCI was attenuated in zebrafish treated with E2f4 inhibitor

**Figure 5:**
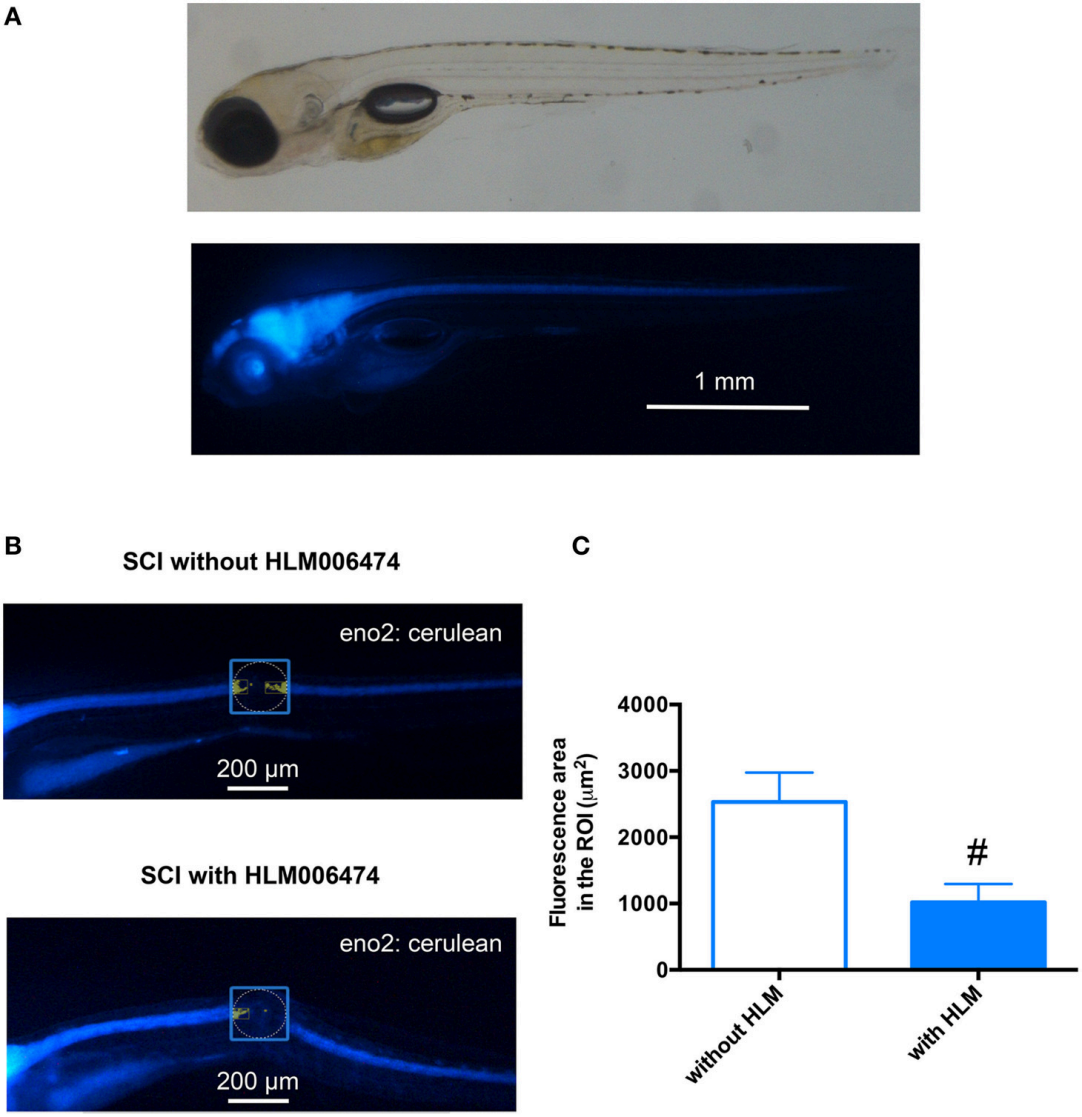
**Neuronal regeneration after SCI was impaired in zebrafish treated with E2F4 inhibitor. (A)** Representative *in vivo* imaging of Tg(eno2: Cerulean) zebrafish. **(B)** Representative *in vivo* imaging of Tg(eno2: Cerulean) zebrafish with SCI at 3 dpi treated with or without HLM006474. **(C)** The Cerulean area in the ROI was quantified and compared between zebrafish with SCI treated with or without HLM006474. *N* = 24 and 22 for zebrafish without and with HLM006474, respectively. ^#^*p* < 0.05 compared to SCI without HLM006474.

We then examined whether the inhibition of e2f4 might attenuate the neuronal regeneration of spinal cord in zebrafish after SCI. We performed *in vivo* imaging of zebrafish selectively expressing a fluorescent protein, Cerulean, in neurons under the control of the *eno2* promoter (Figure 5A). As shown in Figures 5B,C, treatment of HLM006474 significantly reduced the regeneration of neurons at 3 dpi. These results suggest that inhibition of e2f4 attenuates neuronal regeneration after SCI in zebrafish.

## Discussion

### Regulation of zebrafish SCI-specific DEGs through E2F4, TFDP1, and FOXM1

In this study, we demonstrated that e2f4, tfdp1, and foxm1 possibly regulate zebrafish SCI-specific DEGs. E2F4 and TFDP1 are members of the DREAM complex, the master coordinator of the cell cycle (Sadasivam and DeCaprio, 2013). The DREAM complex regulates down-regulation of genes related to mitosis when cells exit the cell cycle and enter into G0 phase in response to differentiation signals or absence of growth factors (Sadasivam and DeCaprio, 2013). When cells leave G0 and enter into the cell cycle, RBL2 dissociates from E2F4 and the MuvB core resulting in the release of the DREAM complex from the promoters of genes related to the cell cycle (Sadasivam and DeCaprio, 2013). When cells enter into the G2/M stage, the MuvB core proteins associate with FOXM1, activating the expression of genes related to the G2/M stage, including CCNA2, CCNB2, NEK2, and PLK1 (Teh et al., 2010; Bella et al., 2014). The expression of these genes was down-regulated in zebrafish SCI at 1 and 3 dpi (Tables S1, S2), suggesting that e2f4 and tfdp1 may be activated and foxm1 may be inhibited after SCI in zebrafish. Genes related to the cell cycle were also significantly enriched in the DEGs specific for zebrafish SCI potentially regulated by at least two of e2f4, tfdp1 and foxm1 at 1 and 3 dpi (Tables S5, S6), suggesting important roles of these TFs in the regulation of DEGs specific for zebrafish SCI.

### Regulation of mouse/rat SCI-specific DEGs

In this study, we demonstrated that Nfic and Tead4 may contribute to the regulation of DEGs specific for mouse/rat DEG. We cannot exclude the possibility, however, that other important factors may regulate the DEGs specific for mammalian SCI. It has been demonstrated that the expression of miR-133b was increased and decreased after SCI in zebrafish (Yu et al., 2011) and rat (Liu et al., 2009), respectively. Connective tissue growth factor (CTGF) is a target of miR-133, and it is down-regulated by miR-133 (Duisters et al., 2009). Furthermore, the expression of CTGF in reactive astrocytes was significantly increased through the down-regulation of miR-133b (Xin et al., 2013), while CTGF expression by reactive astrocytes is associated with matrix deposition and glial scar formation in human cerebral infarction (Schwab et al., 2000). These findings suggest that down-regulation of miR-133b may be responsible, at least in part, for DEGs specific to mammalian SCI. Consistent with this, 19 genes identified as mammalian SCI-specific DEGs at 3 dpi are also predicted to be targets of miR-133b (Wong and Wang, 2015). Further studies are required to elucidate the mechanisms regulating DEGs specific for mammalian SCI.

### Activation of E2F4 is responsible for significant recovery after SCI in zebrafish

We demonstrated that the recovery of locomotive function and neuronal regeneration after SCI were significantly inhibited in zebrafish treated with HLM00646, an E2F4 inhibitor, suggesting that activation of e2f4 after SCI may be responsible, at least in part, for significant recovery in zebrafish. To our knowledge, E2F4 is the only target of HLM00646; however, we cannot exclude the possibility that HLM00646 inhibited zebrafish SCI recovery through targeting molecules other than e2f4. Experiments using *e2f4* knock-down or knock-out zebrafish will confirm the involvement of e2f4 in the significant recovery from SCI in zebrafish. The mechanism of how e2f4 is activated after SCI in zebrafish also remains to be elucidated. It has been demonstrated that E2F4 does not contain a nuclear localization signal, and that the nuclear import of E2F4 is stimulated by association with RBL2 (also called p130) (Moberg et al., 1996). The association between E2F4 and RBL2 is stimulated in response to cell cycle exit (Moberg et al., 1996). It has also been demonstrated that transcriptional repression activity of the DREAM complex is regulated by phosphorylation of the MuvB complex (Sadasivam and DeCaprio, 2013). The MuvB complex is phosphorylated by DYRK1A (Litovchick et al., 2011). Transient expression of DYRK1A in neuronal precursors acts as a binary switch, coupling the end of proliferation and the initiation of neuronal differentiation (Hammerle et al., 2011). These findings suggest that the function of e2f4 may be stimulated by association with rbl2 and activation of dyrk1a after SCI in zebrafish.

The selective activation of e2f4 in zebrafish SCI also suggests that the function of E2F4 may be inhibited in mammalian SCI. It has been demonstrated that 4-hydroxynonenal (HNE), a product of lipid peroxidation, significantly reduced E2F4 protein levels and increased those of the pRb/E2F1 complex (Barrera et al., 2004). E2F4 and E2F1 have opposing roles in regulation of the cell cycle (Crosby and Almasan, 2004). It has also been demonstrated that E2F4 may be transcriptionally repressed by E2F1 (Ma et al., 2004). These findings suggest that the activation of E2F1 may inhibit the function of E2F4 in mammalian SCI. This is consistent with previous reports demonstrating that antioxidant therapies (Bains and Hall, 2012) and inhibition of E2F1 (Wu et al., 2012) confers neuroprotection after SCI in mammals. It has been demonstrated that forced expression of cyclin-dependent kinase inhibitor 1A (CDKN1A) can promote axonal regeneration following SCI in rats (Tanaka et al., 2004). CDKN1A inhibits E2F1 (Ookawa et al., 2001) and activates E2F4 (Benson et al., 2014). These studies suggest that CDKN1A may be inhibited after SCI in mammals, resulting in the activation of E2F1, and inhibition of E2F4. Activation of E2F4 may therefore be a therapeutic strategy after SCI in mammals.

### Screening chemicals and genes for treatment of SCI using larval zebrafish

In this study, we developed a SCI model using larval zebrafish that can be used to assess neuronal regeneration by *in vivo* imaging and functional recovery by behavioral profiling in multi-well-plates. Zebrafish can absorb a wide range of chemicals from the medium in which they swim, and metabolize them in a similar way to mammals (Nishimura et al., 2015a). Zebrafish are also highly amenable to genome editing (Kotani et al., 2015). Phenotype-based screening using zebrafish has successfully discovered novel therapeutic drugs, novel uses for existing drugs and novel disease-related genes (MacRae and Peterson, 2015). Integration of screening using zebrafish and detailed characterization using rodents may minimize risks in extrapolating animal model data to human biology (Sogorb et al., 2014). Behavioral profiling and *in vivo* imaging of neuronal cells using larval zebrafish is a useful strategy for identifying genes and chemicals promoting recovery after SCI.

In summary, we demonstrated that activation of e2f4 may be responsible, at least in part, for significant recovery after SCI in zebrafish. This provides novel insights into the mechanism underlying the poor recovery after SCI in mammals, and into potential therapeutic strategies.

## Author contributions

YN and SS are co-first authors. YN conceived the study, performed comparative transcriptome analysis using systems biology approaches, and wrote the paper. SS performed the zebrafish experiments, analyzed the data, and draw the summary illustration. YH performed zebrafish experiments. SM, YA, MY, SO, KK, and RK provided assistance with experiments. TT conceived the experiments and wrote the paper.

### Conflict of interest statement

The authors declare that the research was conducted in the absence of any commercial or financial relationships that could be construed as a potential conflict of interest.

## Footnotes

^1^Ministry of the Environment Japan, 2014, The Law for the Humane Treatment and Management of Animals, Law No. 105, October 1, 1973, revised May 30, 2014.

^2^Ministry of the Environment Japan, Standards Relating to the Care and Management of Laboratory Animals and Relief of Pain, 2013, Notice No. 88, April 28, 2006, revised August 30, 2013.

^3^Science Council of Japan, 2006, Guidelines for Proper Conduct of Animal Experiments, www.scj.go.jp/ja/info/kohyo/pdf/kohyo-20-k16-2e.pdf

## Supplementary material

Table S1 **DEGs in each SCI model and the overlap at 1 dpi. S1-1**, DEGs in mouse SCI at 1 dpi; **S1-2**, DEGs in rat SCI at 1 dpi; **S1-3**, DEGs in zebrafish SCI at 1 dpi; **S1-4**, DEGs common among mouse, rat, and zebrafish SCI at 1 dpi; **S1-5**, DEGs showing opposite differential expression between zebrafish and mouse/rat SCI at 1 dpi; **S1-6**, DEGs unique in zebrafish SCI at 1 dpi; **S1-7**, DEGs unique in mouse and rat SCI at 1 dpi; **S1-8**, DEGs specific for zebrafish SCI at 1 dpi; **S1-9**, DEGs specific for mouse/rat SCI at 1 dpi.

Table S2 **DEGs in each SCI model and the overlap at 3 dpi. S2-1**, DEGs in mouse SCI at 3 dpi; **S2-2**, DEGs in rat SCI at 3 dpi; **S2-3**, DEGs in zebrafish SCI at 3 dpi; **S2-4**, DEGs common among mouse, rat, and zebrafish SCI at 3 dpi; **S2-5**, DEGs showing opposite differential expression between zebrafish and mouse/rat SCI at 3 dpi; **S2-6**, DEGs unique in zebrafish SCI at 3 dpi; **S2-7**, DEGs unique in mouse and rat SCI at 3 dpi; **S2-8**, DEGs specific for zebrafish SCI at 3 dpi; **S2-9**, DEGs specific for mouse/rat SCI at 3 dpi.

Table S3 **Gene ontologies enriched in DEGs specific for zebrafish or mouse/rat SCI. S3-1** Gene ontologies enriched in DEGs specific for zebrafish SCI at 1 dpi; **S3-2**, Gene ontologies enriched in DEGs specific for zebrafish SCI at 3 dpi; **S3-3**, Gene ontologies enriched in DEGs specific for mouse/rat SCI at 1 dpi; **S3-4**, Gene ontologies enriched in DEGs specific for mouse/rat SCI at 3 dpi.

Table S4 **Transcription factors potentially regulating DEGs specific for zebrafish SCI or mouse/rat SCI. S4-1**, Transcription factors potentially regulating DEGs specific for zebrafish SCI; **S4-2**, Transcription factors potentially regulating DEGs specific for mouse/rat SCI.

Table S5 **DEGs specific for zebrafish or mouse/rat SCI potentially regulated by TFs. S5-1** DEGs specific for zebrafish SCI at 1 dpi potentially regulated by at least two of e2f4, tfdp1, and foxm1; **S5-2**, DEGs specific for zebrafish SCI at 3 dpi potentially regulated by at least two of e2f4, tfdp1, and foxm1; **S5-3**, DEGs specific for mouse/rat SCI at 1 dpi potentially regulated by Nfic and Tead4; **S5-4**, DEGs specific for mouse/rat SCI at 3 dpi potentially regulated by Nfic and Tead4.

Table S6 **Gene ontologies enriched in DEGs potentially regulated by TFs. S6-1** Gene ontologies enriched in DEGs specific for zebrafish SCI at 1 dpi potentially regulated by at least two of e2f4, tfdp1, and foxm1; **S6-2**, Gene ontologies enriched in DEGs specific for zebrafish SCI at 3 dpi potentially regulated by at least two of e2f4, tfdp1, and foxm1; **S6-3**, Gene ontologies enriched in DEGs specific for mouse/rat SCI at 1 dpi potentially regulated by Nfic and Tead4; **S6-4**, Gene ontologies enriched in DEGs specific for mouse/rat SCI at 3 dpi potentially regulated by Nfic and Tead4.

Movie S1 **Cutting the zebrafish spinal cord using a microdissection device**.

Movie S2 **Locomotion of zebrafish with SCI responding to the change from light to dark conditions**.

## Abbreviations

SCI: Spinal cord injury
ECM: extracellular matrix
DEGs: differentially expressed genes
TFs: transcription factors
DREAM complex: TFDP1, RBL2, E2F4 and the MuvB core complex
dpi: days-post-injury
dpf: days-post-fertilization.
